# Tracing local and regional clusters of carbapenemase-producing *Klebsiella pneumoniae* ST512 with whole genome sequencing, Finland, 2013 to 2018

**DOI:** 10.2807/1560-7917.ES.2019.24.38.1800522

**Published:** 2019-09-19

**Authors:** Janko van Beek, Kati Räisänen, Markku Broas, Jari Kauranen, Arja Kähkölä, Janne Laine, Eeva Mustonen, Tuija Nurkkala, Teija Puhto, Jaana Sinkkonen, Senja Torvinen, Tarja Vornanen, Risto Vuento, Jari Jalava, Outi Lyytikäinen

**Affiliations:** 1 Department of Health Security, National Institute for Health and Welfare, Helsinki, Finland; 2 European Programme for Public Health Microbiology Training (EUPHEM), European Centre for Disease Prevention and Control (ECDC), Stockholm, Sweden; 3 Infection-hospital hygiene unit, Lapland Central Hospital, Rovaniemi, Finland; 4 NordLab, Oulu, Finland; 5 Department of Infectious Diseases, Tampere University Hospital and University of Tampere, Faculty of Medicine and Life Sciences, Tampere, Finland; 6 Hospital hygiene and infectious diseases unit, Länsi-Pohja Central Hospital, Kemi, Finland; 7 Department of Infection Control, Oulu University Hospital, Oulu, Finland; 8 Kainuu Central Hospital, Kajaani, Finland; 9 FimLab laboratories, Tampere, Finland

**Keywords:** CPE, Klebsiella pneumoniae, whole genome sequencing, outbreak, hospital, Finland, healthcare-associated infections, bacterial infections

## Abstract

**Background:**

Two epidemiologically-unrelated clusters of *Klebsiella pneumoniae* carbapenemase (KPC)-producing *K. pneumoniae* were detected among several healthcare facilities (HCF) in Finland by routine surveillance using whole genome sequencing (WGS).

**Aim:**

The objective was to investigate transmission chains to stop further spread of the responsible strain.

**Methods:**

In this observational retrospective study, cases were defined as patients with *K. pneumoniae* KPC-3 sequence type (ST)512 strain detected in Finland from August 2013 to May 2018. Environmental specimens were obtained from surfaces, sinks and toilets in affected wards. WGS was performed on *K. pneumoniae* cultures using Illumina MiSeq platform and data were analysed using Ridom SeqShere software *K. pneumoniae* core genome multilocus sequence typing (cgMLST) scheme. Epidemiological information of the cases was provided by HCFs.

**Results:**

We identified 20 cases in six HCFs: cluster 1 included 18 cases in five HCFs and cluster 2 two cases in one HCF. In cluster 1, a link with a foreign country was unclear, 6/18 cases without overlapping stay had occupied the same room in one of the five HCFs within > 3 years. In cluster 2, the index case was transferred from abroad, both cases occupied the same room 8 months apart. A strain identical to that of the two cases in cgMLST was isolated from the toilet of the room, suggesting a clonal origin.

**Conclusions:**

The clusters were mostly related to case transfer between facilities and likely involved environmental transmission. We show that CPE surveillance using WGS and collaboration between hospitals are crucial to identify clusters and trace transmission chains.

## Introduction

Carbapenemase-producing Enterobacteriaceae (CPE) pose a notable threat to patients and healthcare systems in Europe and globally [1-3]. Infections caused by CPE are associated with high mortality, primarily due to delays in administration of effective treatment and the limited availability of treatment options. CPE are predominantly hospital-acquired and carbapenem resistance is transmitted via clonal spread or horizontal plasmid-mediated transmission [4,5]. Whole genome sequencing (WGS) has recently become the gold standard for bacterial typing and has previously shown to be useful to trace back transmission chains and reveal unexpected transmission routes within outbreaks [6-9].

Inter-regional spread or an endemic situation of CPE was reported by 13 of 38 (34%) European countries in 2015 [10]. Currently CPE are endemic in Greece, Italy and Turkey, posing a threat of cross-border spread during international patient transfers [3]. CPE are rare in Finland and have been detected sporadically, mostly related to hospitalisation abroad [11]. Only one outbreak has been detected so far when nine patients were found colonised by *Klebsiella pneumoniae* carbapenemase (KPC)-producing *K. pneumoniae* (KPC-KP) in a primary care hospital in Southern Finland in 2013 [12].

Here we describe how we traced back transmission chains in a single hospital cluster with two *K. pneumoniae* KPC-3 sequence type (ST)512 patients in Western Finland, and the first regional cluster with 18 *K. pneumoniae* KPC-3 ST512 patients in five healthcare facilities (HCF) in Northern Finland using WGS. We also report the results of patient screening and environmental investigations related to these clusters.

## Methods

### Population and surveillance

In Finland (population 5.5 million in 2018), publicly funded healthcare is organised in 20 geographically and administratively defined healthcare districts with a population range from 44,000 to 1.6 million inhabitants. Secondary care is provided by 15 central hospitals and five university hospitals additionally provide tertiary care services. Primary care is provided locally by public healthcare centres.

All Finnish clinical microbiological laboratories electronically notify all isolates with reduced susceptibility to carbapenems to the National Infectious Disease Registry and send bacterial strains to the Expert Microbiology Unit of the National Institute for Health and Welfare for confirmation and national surveillance. Since 2015 all CPE strains have routinely been sequenced using WGS and older isolates (2009–14) have been sequenced retrospectively.

### National guidelines for control of multidrug-resistant bacteria

The national guidelines for controlling multidrug-resistant (MDR) bacteria cover CPE [13]. According to the guidelines all high-risk patients (defined as patients who either were transferred from a foreign hospital or hospitalised abroad during the previous year) are placed in single rooms, managed by contact precautions like CPE-positive patients, and screened (twice 24 hours apart) for MDR bacteria at the time of admission. When CPE patients are newly identified, potentially exposed contact patients, such as current roommates, are screened and those exposed patients who are already discharged are flagged in the patient’s records to institute infection control measures and screening in case of re-admission. More extensive screening for CPE, like point prevalence surveys on wards or units, are considered if positive patients are found during the contact screening. When patients are transferred to other facilities, receiving facilities are informed about the patient’s infection or colonisation caused by CPE.

### Case definition and epidemiological investigation

Based on WGS national surveillance analysis, cases were defined as patients with specimens dated from August 2013 onwards with a positive culture of *K. pneumoniae* KPC-3 ST512 belonging to one of two whole genome sequence clusters (GenBank accession numbers cluster 1 and 2: SAMN10791377 and SAMN10791378, respectively) with 10 or less allele differences in core genome multilocus sequence typing (cgMLST) analysis. This cut-off was experimentally determined and has been used in similar studies previously [14]. National (public health microbiologists and epidemiologists) and regional (clinical microbiologists, infection control nurses and infectious disease specialists) experts formed the outbreak investigation team. Background information (hospital stay, ward specialty, underlying disease/condition, antimicrobial treatment, clinical outcome, hospital transfers, and travel history) of cases, and patient and environmental screening results were provided by the respective HCF (HCF A: 615 beds; HCF B: 151 beds; HCF C: 113 beds; HCF D: 218 beds; HCF E: 40 beds; HCF F: 779 beds). Data were analysed using descriptive statistics.

### Patient and environmental microbiological testing

Environmental specimens (surfaces, sinks, toilets) were taken with sterile, cotton-tipped swabs. Patient and environmental specimens were plated on selective chromogenic KPC plates (CHROMagar, Paris, France) to screen for MDR Enterobacteriaceae. Isolates were characterised by matrix-assisted laser desorption/ionisation time-of-flight (MALDI-TOF) mass spectrometry (VITEK MS, bioMeriéux, Marcy-L’Etoile, France), and antimicrobial susceptibilities were assessed by disk diffusion method according to clinical breakpoints as published by the European Committee on Antimicrobial Susceptibility Testing [15], or by gradient minimum inhibitory concentrations (MIC) determination test (Etest, bioMeriéux, Marcy-L’Etoile, France). The carbapenemase gene was determined by multiplex real-time PCR for isolates with reduced susceptibility to any carbapenem as per the national guideline [13,16].

### Whole genome sequencing

WGS was implemented for all CPE positive strains. Sequencing was performed on a MiSeq instrument (Illumina, San Diego, California (CA), United States (US)). For the library preparation 1 ng purified DNA was used with a NexteraXT V2 DNA sample preparation kit (Illumina) and paired-end sequenced with a 2 × 150 bp kit (Illumina). Libraries were scaled to reach 100-fold coverage. Read mapping analysis was performed from the FASTQ files. Files were transferred to the Centre for Scientific Computing (CSC) environment where sequences were trimmed with Trimmomatic (version 0.33), quality was verified with FastQC (version 0.11.6), resistance genes and multilocus sequence typing (MLST) were analysed with SRST2 version 0.2.0 [17,18]. Commercial software SeqSphere + (Ridom GmbH, Münster, Germany) and the available cgMLST scheme for *K. pneumoniae* from Ridom, were used to prepare the minimum spanning tree.

### Ethical statement

The Finnish Ministry of Social Affairs and Health determined that no ethics committee review was required for this study since it was conducted as part of an investigation in response to an acute public health problem. Collection of CPE strains is based on the communicable disease law.

## Results

### Outbreak detection

**Figure 1:**
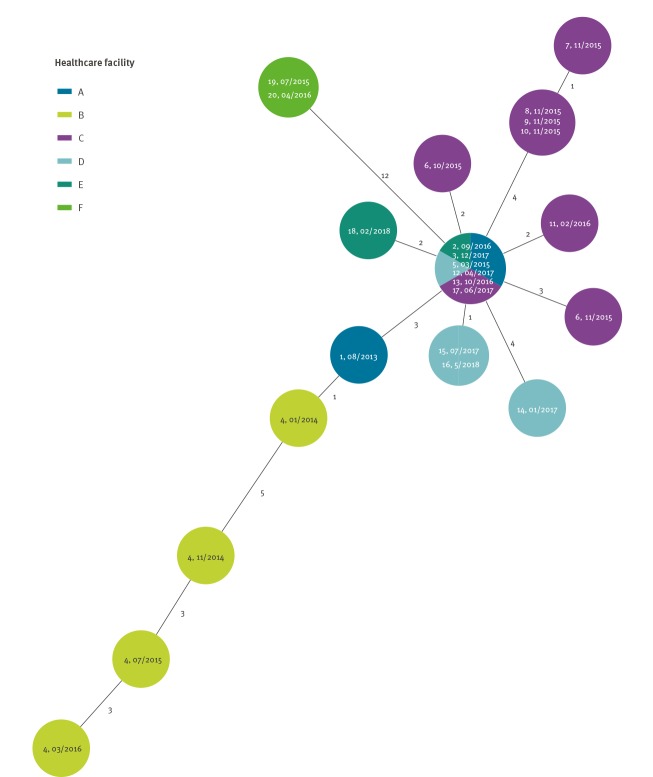
Minimum spanning tree of core genome sequences of *Klebsiella pneumoniae* carbapenemase-3 producing *K. pneumoniae* ST512 isolated from cases, Finland, 2013–2018 (n = 20 cases)

**Figure 2:**
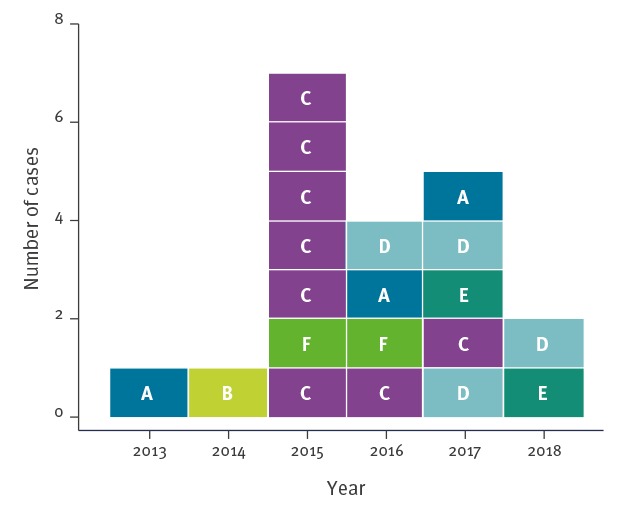
Distribution over time of *Klebsiella pneumoniae* carbapenemase-3 producing *K. pneumoniae *ST512 cases, Finland, 2013–2018 (n = 20 cases)

**Figure 3:**
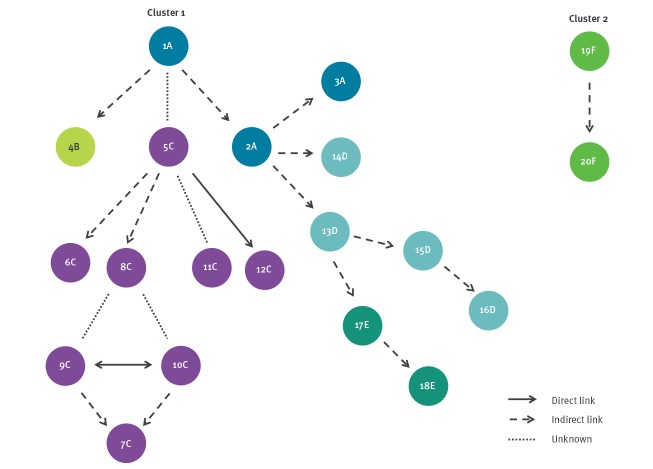
Epidemiological links between *Klebsiella pneumoniae* carbapenemase-3 producing *K. pneumoniae* ST512 cases, Finland, 2013–2018 (n = 20 cases)

Two separate clusters of closely related (less than 10 allele difference) core genome sequences of *K. pneumoniae* KPC-3 ST512 were identified in a minimum spanning tree analysis of the national surveillance data (Figure 1). The first cluster included 18 cases distributed regionally over five HCFs (HCF A–E) in Northern Finland between August 2013 and May 2018 (Figure 2 and 3). The second cluster included two cases in HCF F between July 2015 and April 2016. The causative strain of the first cluster was only susceptible to ceftazidime/avibactam, colistin and gentamicin and the strain of the second cluster to gentamicin and colistin (not tested to ceftazidime/avibactam).

ST: sequence type.

Each circle (node) represents one or multiple identical sequences. The number between the nodes indicates the number of allele differences. The text in each circle indicates the case identifier, sample month/sample year, and the colour indicates the healthcare facility (A–F).

ST: sequence type.

Letters inside boxes indicate the healthcare facility (A–F).

ST: sequence type.

Nodes represent cases (1–20) and letters healthcare facilities (A–F). Arrows indicate epidemiological link between cases, directly (patient-to-patient) or indirectly (environmental source or via undetected intermediate patient).

Bidirectional arrows between two cases indicate that one case could have transmitted the strain to another or vice-versa.

### Description of cases and transmission chains

The median age of the 20 cases was 69 years (range: 30–89 years), 15 were male, and all but two had at least one underlying disease. Sixteen cases were found through positive results in clinical specimens (i.e. specimens requested by the attending physician due to clinical signs and symptoms of the patient) and four in routine or enhanced screening. Eight cases received antimicrobials for a clinical infection caused by *K. pneumoniae* KPC-3 ST512 and one patient died attributable to the infection. A summary of patient information is presented in the Table.

**Table:** Characteristics of cases with *Klebsiella pneumoniae* carbapenemase-3 producing *K. pneumoniae* ST512 by healthcare facility and first positive specimen, Finland, 2013–2018 (n = 20 cases)

ID	HCF	Level of care	Ward specialty	Underlying disease/condition	Month-year of specimen collection	Specimen type	Indication	Epidemiological link
1	A	Tertiary	Surgery	Trauma, drug abuse	Aug-13	Abdominal fluid	Clinical	Unclear
2	A	Tertiary	Surgery	Hypertension, CHD, carcinoma	Sep-16	Liver surface	Clinical	Common room in HCF A with case 1 (no overlapping stay)
3	A	Tertiary	Surgery	Pancreatitis	Dec-17	Peritoneal secretion	Clinical	Common room in HCF A with case 1 and 2 (no overlapping stay)
4	B	Secondary	Surgery	Cholecystitis	Jan-14,	Blood	Clinical	Preceding stay in HCF A and common room with case 1 (no overlapping stay)
					Nov-14,	Urine	Clinical	
					Jul-15	Urine	Clinical	
					Mar-16	Urine	Clinical	
5	C	Secondary	Internal medicine	Kidney transplantation, haemodialysis	Mar-15	Wound secretion	Clinical	Unknown link to HCF A
6	C	Secondary	Internal medicine	RI, gout	Oct-15,	Urine	Clinical	Same ward X in HCF C with case 5 (no overlapping stay)
					Nov-15	Urine	Screening	
7	C	Secondary	Surgery	Asthma, hypertonia	Nov-15	Urine	Clinical	Common room in ward Y in HCF C with case 9 and 10 (no overlapping stay)
8	C	Secondary	Intensive care	Kidney transplantation, DM, CI	Nov-15	Urine	Screening	Same ward X in HCF C with case 5 (overlapping stay)
9	C	Secondary	Surgery	CHD, CI	Nov-15	Faeces	Screening	Common room in ward Y in HCF C with case 7 (no overlapping stay) and 10 (overlapping stay)
10	C	Secondary	Surgery	Carcinoma	Nov-15	Faeces	Screening	Common room in ward Y in HCF C with case 7 (no overlapping stay) and 9 (overlapping stay)
11	C	Secondary	Surgery	CI, dementia, hypertension	Feb-16	Urine	Clinical	Unknown link to cases in HCF C
12	C	Secondary	Internal outpatient	Kidney transplantation, haemodialysis	Apr-17	Urine	Clinical	Weekly haemodialysis in HCF C with case 5
13	D	Secondary	Surgery	Alcoholism, chronic pancreatitis, DM	Oct-16	Urine	Clinical	Preceding stay in HCF A and common room with cases 1, 2 and 4 (no overlapping stay)
14	D	Secondary	Emergency room	Carcinoma, hypothyroidism, obesity	Jan-17	Urine	Clinical	Preceding stay in HCF A and common room with case 1, 2 and 4 (no overlapping stay)
15	D	Secondary	Surgery	Diverticulosis, DM, hypertension	Jul-17	Abscess	Clinical	Common room in HCF D with case 13 (no overlapping stay)
16	D	Secondary	Internal medicine	CHD, COPD, RI	May-18	Urine	Clinical	Common room in HCF D with case 15 (no overlapping stay)
17	E	Primary	General medicine	Carcinoma, COPD, DM, IC	Jun-17	Urine	Clinical	Preceding stay in HCF D and common room with case 13 (no overlapping stay)
18	E	Primary	General medicine	Obesity, recurrent erysipelas	Feb-18	Urine	Clinical	Common room in HCF E with case 17 (no overlapping stay)
19	F	Tertiary	Cardiac surgery	No	Jul-15	Faeces	Screening	Hospital stay in Italy
20	F	Tertiary	Cardiac surgery	Necrotising fasciitis, empyema	Apr-16	Wound	Clinical	Common room in HCF F with case 19 (no overlapping stay)

CHD: coronary heart disease; CI: cardiac insufficiency; COPD: chronic obstructive pulmonary disease; DM: diabetes mellitus; HCF: healthcare facility; IC: insufficientia cordis; ID: identifier; RI: renal insufficiency; ST: sequence type.

#### Cluster 1

The index case (case 1) of the first cluster was a patient with trauma and a drug abuse history. Recent travel history abroad was unclear. This case was treated in HCF A and detected positive by a clinical specimen in August 2013. Case 1 had previously been found negative during the same hospitalisation period when screened for MDR bacteria at intensive care unit (ICU) admission in July 2013 and also weekly according to the routines surveillance cultures of the ICU. Cases 2 and 3 in HCF A were found by positive clinical specimens in September 2016 and December 2017 and had been treated 33 months and 50 months later in the same room as case 1 after the ICU care, respectively.

The strain spread to HCF B via case 4 who had had a preceding hospitalisation in HCF A in the same room, without overlapping stay, as case 1 and was subsequently transferred to HCF B. Case 4 remained positive for the outbreak strain for more than 2 years, but no further transmission was detected.

Case 5, the first one in HCF C, was identified by a positive clinical specimen in March 2015. No direct epidemiological link between case 5 and any previous cases or HCF A was found. Case 6 in HCF C was identified by a positive clinical specimen 6 months after case 5 and likely obtained the strain via case 5 as they both were treated in ward X even though there was no overlapping stay or common room. Cases 8, 9 and 10 in HCF C were identified by ward screening in November 2015. Case 8 likely obtained the strain via case 5 since both were treated in ward X with overlapping stay, though they did not share a common room. Based on the sequence analysis, cases 9 and/or 10 likely obtained the strain from case 8. Cases 9 and 10 had shared a room in ward Y, which had also been occupied by case 7 but without overlapping stay. Although case 7 was identified by a positive clinical specimen four days before cases 8, 9 and 10, sequence analysis revealed that case 7 most likely obtained the strain via case 9 or 10. Case 11 in HCF C was identified by a clinical specimen in February 2016 and had a strain with a sequence most likely derived from case 5, but no epidemiological link between these cases was found. Case 12 in HCF C was identified by a clinical specimen in April 2017 and had a strain with an identical sequence as case 5. Both cases (case 5 and 12) had attended weekly haemodialysis in HCF C. Case 12 was previously found negative in the screening conducted after case 5 was found, but thereafter extended-spectrum beta lactamases (ESBL)-producing bacteria were detected several times in clinical specimens before the detection of *K. pneumoniae* KPC-3 ST512.

The strain spread to HCF D by cases 13 and 14 who were identified by clinical specimens in October 2016 and January 2017 respectively. Both cases had stayed in the same room as cases 1, 2, and 4 in HCF A (June 2016 to September 2016 and October 2016, respectively) and were subsequently transferred to HCF D. Case 15 in HCF D was identified by a positive clinical specimen in July 2017 and most likely got the strain via case 13 in the same ward who occupied the same room but without overlapping stay. Case 16 in HCF D was identified in May 2018. This case had a strain with an identical sequence as case 15, and a preceding hospitalisation in HCF D in the same ward and common room as case 15 but without overlapping stay.

The strain was introduced to HCF E in June 2017 by case 17 (found by a positive clinical specimen) who had a strain with an identical sequence and had a preceding hospitalisation in HCF D in the same ward and in a common room as case 13 but without overlapping stay. Case 17 was subsequently transferred to HCF E. Case 18 in HCF E was identified in February 2018 by a positive clinical specimen and obtained the strain in HCF E via case 17 in the same ward who occupied the same room but without overlapping stay.

The median time interval between the first positive specimens of all cases in cluster 1 was 3.0 months (range: 0–15 months).

#### Cluster 2

The second cluster consisted of two cases (case 19 and 20) identified in HCF F with an identical sequence and with 12 allele differences compared with the nearest strain of the regional cluster in Northern Finland (Table, Figure 1, 2 and 3). Case 19 was transferred from an Italian hospital and found positive by routine screening, case 20 by a clinical specimen. Both cases occupied the same room more than 8 months apart.

### Enhanced screening of patients and environment

After identification of the first case (case 5) in HCF C, 17 specimens of nine potentially exposed patients were tested and all were negative. After identification of the second case in HCF C (case 6), around 200 potentially exposed patients were screened in November 2015 and three new cases (case 8, 9 and 10) were found. The screening included discharged patients and two screening rounds in the haemodialysis unit and in one ward. Another ward was closed down for a week in December 2015 and 29 environmental specimens were subsequently taken, but all tested negative. 

After detection of the third case in HCF D (case 15) 32 potentially exposed patients were screened and 16 environmental specimens were obtained in August 2017, all patient and environmental specimens tested negative. 

HCF E screened 29 patients after detection of their second case (case 18) in March 2018 and obtained 46 environmental specimens, but all tested negative. 

HCF A screened 26 patients and obtained 16 environmental specimens in March 2018 when the room occupied by several cases (case 1, 2, 3, 4, 13 and 14) was identified. All specimens tested negative.

After identification of the second case in HCF F (case 20) in April 2016, 54 potentially exposed patients treated in the same room as case 19 and 20 were flagged and specimens could be obtained from 28 of them; all tested negative. The hospital staff also performed two cross-sectional screening rounds in the same ward (n = 32), and obtained 142 environmental specimens. Seven environmental specimens tested positive for *K. pneumoniae* KPC-3 ST512, and were either identical or showed maximum three alleles differences compared with the strains found in specimens obtained from cases 19 and 20 (data not shown). The first specimens were obtained after ordinary cleaning (no disinfectant) of the patient room. The positive sites were the patient desk, windowsill, floor drain, toilet seat, and inner toilet surface nearby the water edge. Contact isolation and cleaning (peracetic acid/hydrogen peroxide) were performed in the room. In the control specimens the inner toilet surface remained positive and the sink trap in the room was a new positive site. Terminal cleaning (peracetic acid/hydrogen peroxide) was performed after the patient discharge, also thereafter the inner toilet surface remained positive and the floor drain was again positive. The toilet remained positive even after treatment with NOCOSPRAY (OXY’PHARM, Paris, France) and with 1,000 ppm chlorine. Finally, it was negative after 2,000 ppm chlorine treatment and also in three control specimens (twice in May 2016 and once in March 2017).

## Discussion

Here we describe two clusters of KPC-KP: one single hospital cluster with two cases and one regional cluster with 18 cases spread in five HCFs. Transfer of unknown carriers between HCFs and environmental contamination seemed to play a key role in KPC-KP transmission. The regional spread was initially not recognised due to transfer of previously unknown positive patients, long time intervals between successive cases, and distribution of cases in several HCFs.

The two clusters mainly affected acute care hospitals and most of the cases were detected by clinical specimens. Extensive screening of potentially exposed contact patients only identified a few new cases. Many of the patients were not only colonised by KPC-KP but had clinical infections, and one death was related to infection caused by KPC-KP. It also turned out that the disinfection control methods used previously were not effective in controlling the spread. The regional cluster is still ongoing in one HCF despite extensive measures including isolation of cases, screening, and enhanced environmental cleaning.

*K. pneumoniae* ST258, and related strains like ST512 with KPC carbapenemase gene as detected in this study, have caused healthcare-associated outbreaks globally since they were discovered in early 2000 and are considered to be endemic in HCFs in countries like China, Colombia, Greece, Israel, Italy, Puerto Rico, and the US [19,20]. In Finland, the first *K. pneumoniae* with KPC-2 gene was found in 2009 in a patient repatriated from Greece [21]. The first transfer of KPC-KP (ST258, KPC-2) between two patients in Finland was confirmed in 2009 (data not shown) and the first outbreak caused by KPC-KP (ST512, KPC-3) occurred in 2013 [12]. In the previous outbreak, no direct link to travel abroad or environmental contamination was detected. The outbreak was successfully controlled by rapid and extensive screening together with cohorting and isolating carriers and exposed patients.

The core genome sequence analysis showed two distinct clusters with 12 allele differences, and we interpreted that both clusters were separate outbreaks. However, case 4 acquired 11 allele differences over the course of 2 years and 3 months, showing that analysis of WGS data using a strict cut-off needs to be complemented with epidemiological data for correct interpretation (as was done in this study). There was no patient transfer between HCFs of the two regions and the index case of the single hospital cluster was transferred from a hospital in Italy where KPC-KP is endemic. The origin of the regional cluster remained unknown. It is possible that undetected cases causing environmental contamination have previously existed. Notably, the index patient had negative surveillance cultures for MDR microbes before ward care during intensive care.

We found an epidemiological link (travel history, common ward or room) for most of the cases (16/20). It is likely that we missed cases since epidemiological links for four cases were not found. It is unknown how the strain was transmitted to HCF C since we could not find an epidemiological link between the first case (case 5) of HCF C and other HCFs. We know that three patients were transferred from HCF A to HCF C before case 5 was detected. One of these three patients had shared a room with case 5, this might have caused transmission between facilities (data not shown). This could not be verified since the patient died before the screening was performed.

Only two cases with overlapping stays in a common room were found in these clusters, supporting our hypothesis of environmental contamination and subsequent transmission to new cases. Outbreaks of *K. pneumoniae* with a persisting environmental reservoir and high resistance to cleaning efforts and as shown in HCF F in this study have been previously reported [7,22-27]. Both cases of the single hospital cluster stayed in the same room more than 8 months apart, and the inanimate surfaces in the room and bathroom were found repeatedly positive, even after cleaning and decontamination. Although we think that most of the environmental contamination was caused by the second case since the specimens were taken after this case had stayed in the room, the long interval between the two cases and the efforts required for the decontamination suggest that the environment was the source of KPC-KP transmission. An alternative hypothesis could be that cases in this study did not obtain the strain from the environment, but directly from unknown carriers or indirectly via hands of healthcare workers.

Six cases could be linked to a common room in HCF A without overlapping stays within a period of 3 years and 1 month. This suggests that environmental transmission might have been the main transmission route in the regional cluster as well. Environmental specimens were obtained twice from this room, 2 months after the last positive case and tested negative, which could be a sign that the room has now been decontaminated. We, however, recommend to keep monitoring the room to circumvent the risk of false negative test results.

Both clusters were recognised by national surveillance using WGS, and stress the relevance of national CPE surveillance. Multiple interventions are needed to contain further spread. We could learn from Israel which has implemented and published a national strategy to contain a large outbreak of CPE [28]. Regional coordination needs to be implemented to actively monitor colonisation status of existing cases and trace-back transmission routes of new cases, especially when there is no link to hospitalisation abroad. Like our colleagues in Israel, we recommend that rooms occupied by CPE-positive patients be cleaned and disinfected once a day with dedicated, single-patient or single-use equipment [29]. Special attention should be paid to terminal cleaning following discharge, even to perform it twice by different teams and with different equipment. This applies to all CPE-positive patients, not only in outbreaks and independently of the test results of environmental specimens (positive/negative). If there is no link to a foreign country, the trace-back period for epidemiological investigations should be long enough to ensure finding all links. Sometimes more than 1 year may be needed. Since the yield in screening potentially exposed contact patients has constantly been small, we consider starting systematic screening of patients discharged from affected wards as a new strategy. The regional outbreak team needs to be supported by real-time genomic sequencing by the national reference laboratory and a regular feedback communication mechanism to the HCFs is required to stop further transmission.
